# Case report and review of the literature: rare fetus-in-fetu presenting as oropharyngeal epignathus

**DOI:** 10.3389/fsurg.2023.1122327

**Published:** 2023-05-03

**Authors:** Daniel Runggaldier, Michael Reinehr, Hergen Friedrich, Georg Henze, Dominic Good, Claudine Gysin

**Affiliations:** ^1^ Division of Pediatric Otolaryngology, University Children's Hospital, Zurich, Switzerland; ^2^ Department of Otorhinolaryngology, Head & Neck Surgery, University Hospital Zürich, University of Zurich, Zürich, Switzerland; ^3^ Institute for Pathology and Molecular Pathology, University Hospital Zurich, Zurich, Switzerland; ^4^ Department of Anesthesia, University Children’s Hospital, Zürich, Switzerland

**Keywords:** heteropagus, asymmetric twin, fetus in fetu (FIF), neonate airway, epignathus

## Abstract

An epignathus is caused by a continuous spectrum of masses of the oral cavity or oropharynx ranging in its entity from mature teratoma to the exceedingly rare fetus-in-fetu. Due to its location, regardless of the entity, the occurrence of an epignathus is frequently associated with life threatening airway obstruction. Here we demonstrate a case of a fetus-in-fetu presenting as an epignatus. We describe its successful management and review the available literature. Early diagnosis and knowledge of the preoperative workup are essential to enable a multidisciplinary management. Once the airway is secured, surgical excision is the treatment of choice often resulting in a good clinical outcome and prognosis.

## 1. Introduction

Terminology regarding heteropagus (parasitic twins) and its various subtypes is complex with terms often overlapping or being inconsistently used. Heteropagus, with an incidence of less than 0.1 per 100,000, is defined as conjoined twins, in which a severely defective and dependent twin (parasite) is connected to its mostly intact twin (autosite) (1). So far, the wide spectrum of clinical subtypes of heteropagus can be subdivided into endo- or exoparasitic twins. Exoparasitics twins include for instance the epigastric heteropagus, omphalo- and thoracopagus as well as the twin reverse arterial perfusion (TRAP) sequence. On the other hand, endoparasitic twins or so called fetuses-in-fetu are malformed parasitic twins that are usually found within the body of the living partner (2). They are mostly located in the retroperitoneal space (3) but can also present as a mass attached to palatal or pharyngeal structures, which is known as epignathus (4, 5).

Epignathus itself is a heterogenous group of oropharyngeal masses with an incidence of 1 in 35,000 to 1 in 200,000 births and it can be caused by a broad spectrum of entities ranging from mature teratoma to a real fetus-in-fetu (4, 6). However, even by using a broader definition of fetus-in-fetu, the manifestation of fetus-in-fetu as an epignathus is an extremely rare event with only few reports published so far (4, 5). Moreover, the existence of epignathus is linked to fatal airway obstruction, making early diagnosis and subsequent interdisciplinary management including radical surgical excision essential (4, 6).

In the following we report the case of a fetus-in-fetu presenting as epignathus and discuss the interdisciplinary management of the case as well as the available literature.

## 2. Case description

**Figure 1:**
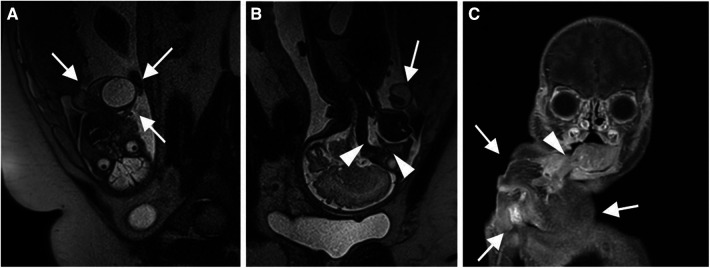
(**A**) Sagittal section (resp. coronal section from perspective of neonate) of T2 weighted MRI depicting the presence of a large epignathus attached to neonate's right oral cavity (white arrows) (B) Coronal section (resp. sagittal section from perspective of neonate) of T2 weighted MRI demonstrating a free upper airway of the neonate (white arrowheads). Epignathus labeled with a white arrow. (C) Coronal section of T1 weighted MRI demonstrating the attachment of the epignathus (white arrows) to the right lateral margin of the tongue (white arrowhead).

**Figure 2:**
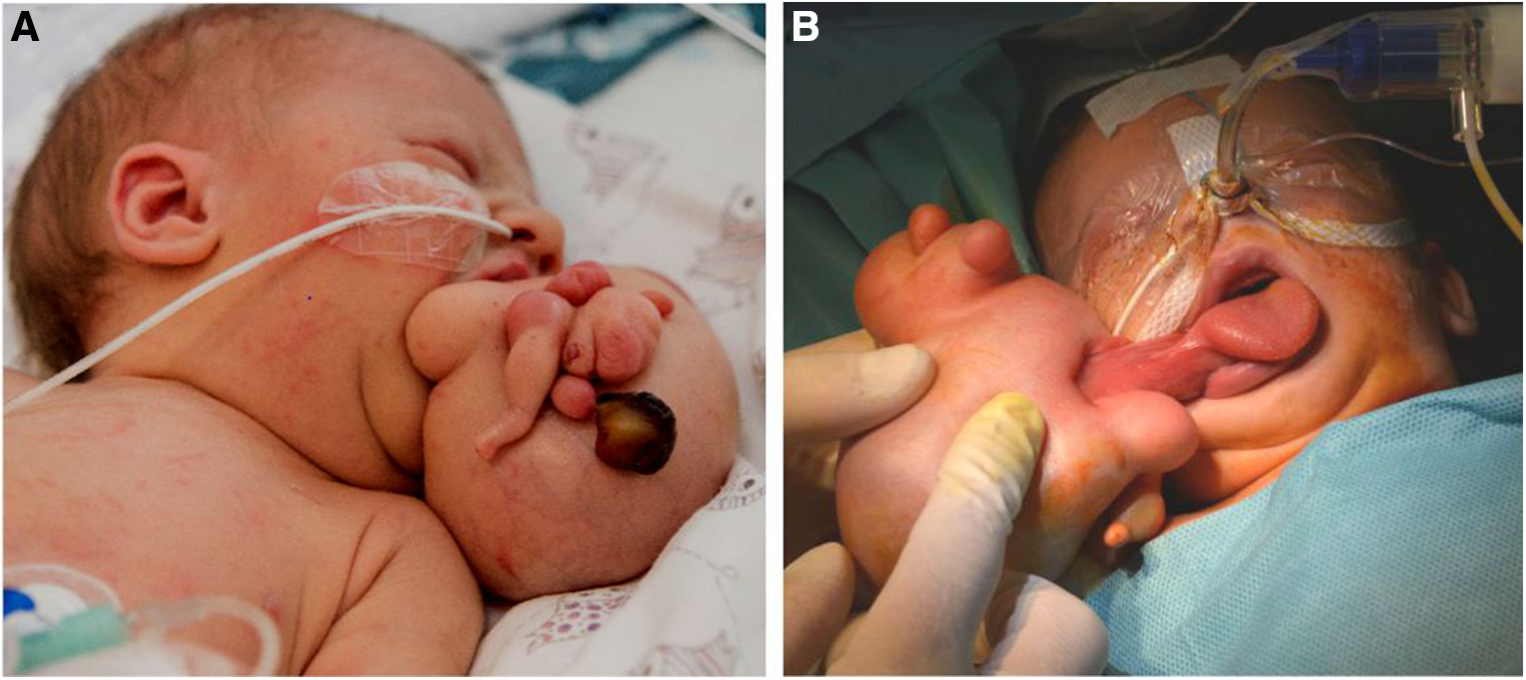
(**A**) Pedunculated and with skin covered mass with multiple round and nodular extensions. (**B**) Attachment to the lateral margin of the tongue and palatal region on the right sinde.

A 29-year-old woman had her second pregnancy. The family history of both Caucasian parents had been unremarkable except of the documentation of a stillborn child. Apart from insulin dependent gestation diabetes the beginning of the pregnancy had been without any noticeable medical events. Exposure to teratogenic agents was denied. In gestation week 30, a routine ultrasound examination was performed leading to the diagnosis of a large tumor originating from the oral cavity with a diameter of 6–7 cm and cystic and solid parts. The main differential diagnosis at that point was a teratoma/epignathus. For further specification, a subsequent fetal MRI scan led to the diagnosis of a large epignathus originating from the right palate and masticator space (Figure 1A). The airways were patent (Figure 1B), so a cesarian section without Ex Utero Intrapartum Treatment (EXIT) procedure was planned at gestation week 38 with an interdisciplinary team including ENT and anesthesiology on standby. The delivery of the full-term male newborn went uneventful with an Apgar Score 8-9-9. Anthropometric measurements of the neonate were as follows: weight 2,945 g (P10–25), length 46.5 cm (<*P*3), head circumference 30 cm (*P* < 3). At all times, the respiration was without restraints and oxygen saturation as well as blood gas analysis were within the normal range. Upon physical examination, a 12 cm × 8 cm × 5 cm stalked fetiforme mass protruding out of the mouth was observed (Figure 2A). Otherwise, the neonate appeared well nourished and healthy. There was no cleft lip. Abdominal and cranial ultrasound examination as well as echocardiography of the neonate were unremarkable except for a small atrial septal defect (ASD II). A postnatal MRI demonstrated the attachment of the fetiforme mass to the soft palate with extension to the masseter muscle and the parotid gland on the right side (Figure 1C). Vascular supply was found to be provided by branches of the right carotid artery. Left facial side and intracranial structures appeared normal in the MRI scan. On the second day of life, excision of the epignathus was performed. At surgery, the epignathus was found to be attached to the floor of the mouth, the lateral margin of the tongue and the soft palate via a structure that resembled macroscopically an umbilical cord (Figure 2B). Incision of the mucosa revealed two veins and one artery as well as a cystic structure, which could be ligated and detached in the depth. The large epignathus could be removed together with the right part of a pairwise arranged uvula and sent for histopathological analysis.

**Figure 3:**
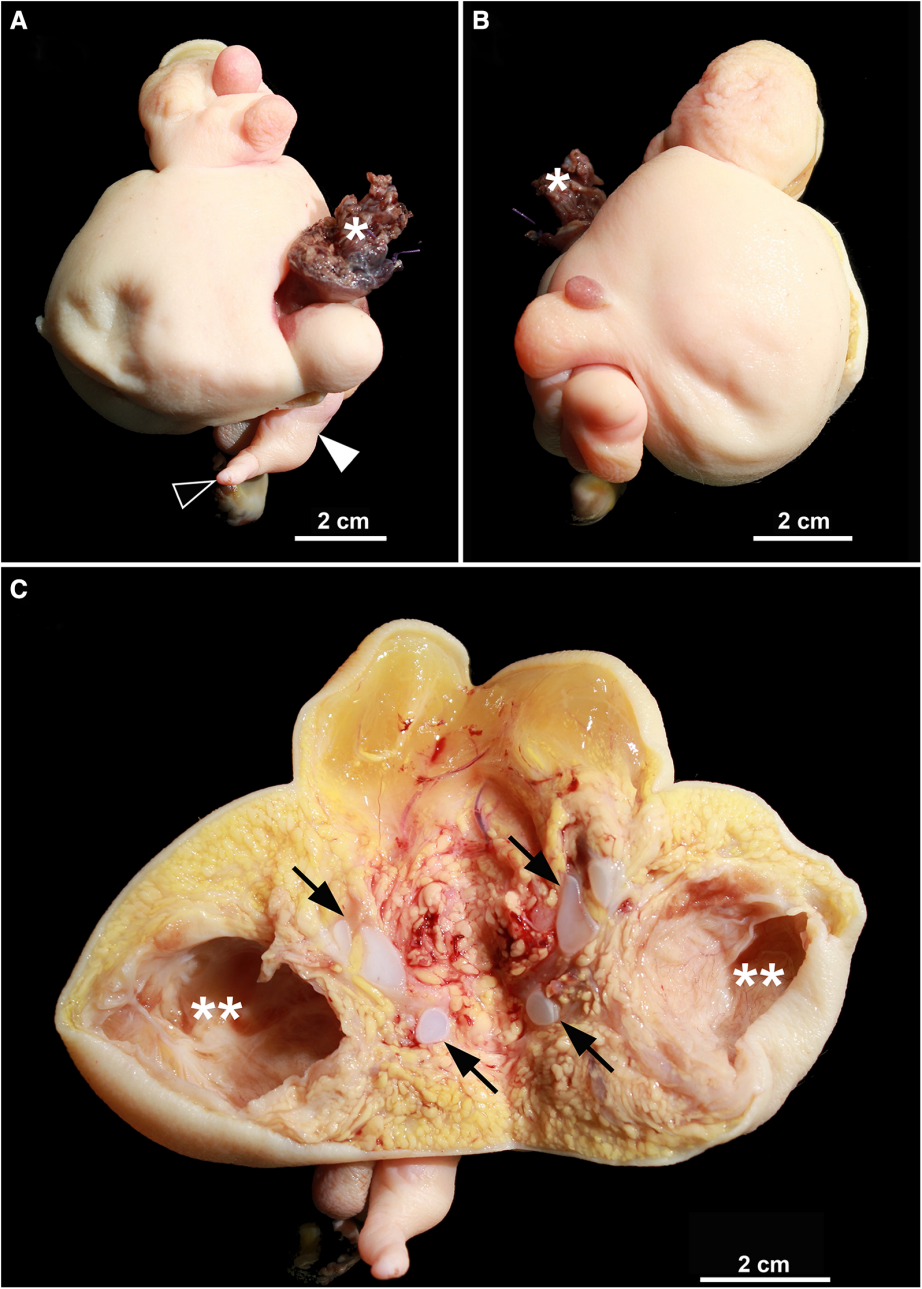
(**A,B**) excision of the fetiform mass in external view. White asterisk marks the peduncle and the resection margin, respectively. White arrowhead shows the rudimentary leg, black arrowhead the single toe. (**C**) Frontal section, unfolded. Abundant adipose tissue, some of which is well perfused, is visible. Black arrows mark rudimentary cartilaginous vertebral anlagen. White double asterisk shows the cerebral vesicle. Other formed organs are not macroscopically delineated.

**Figure 4:**
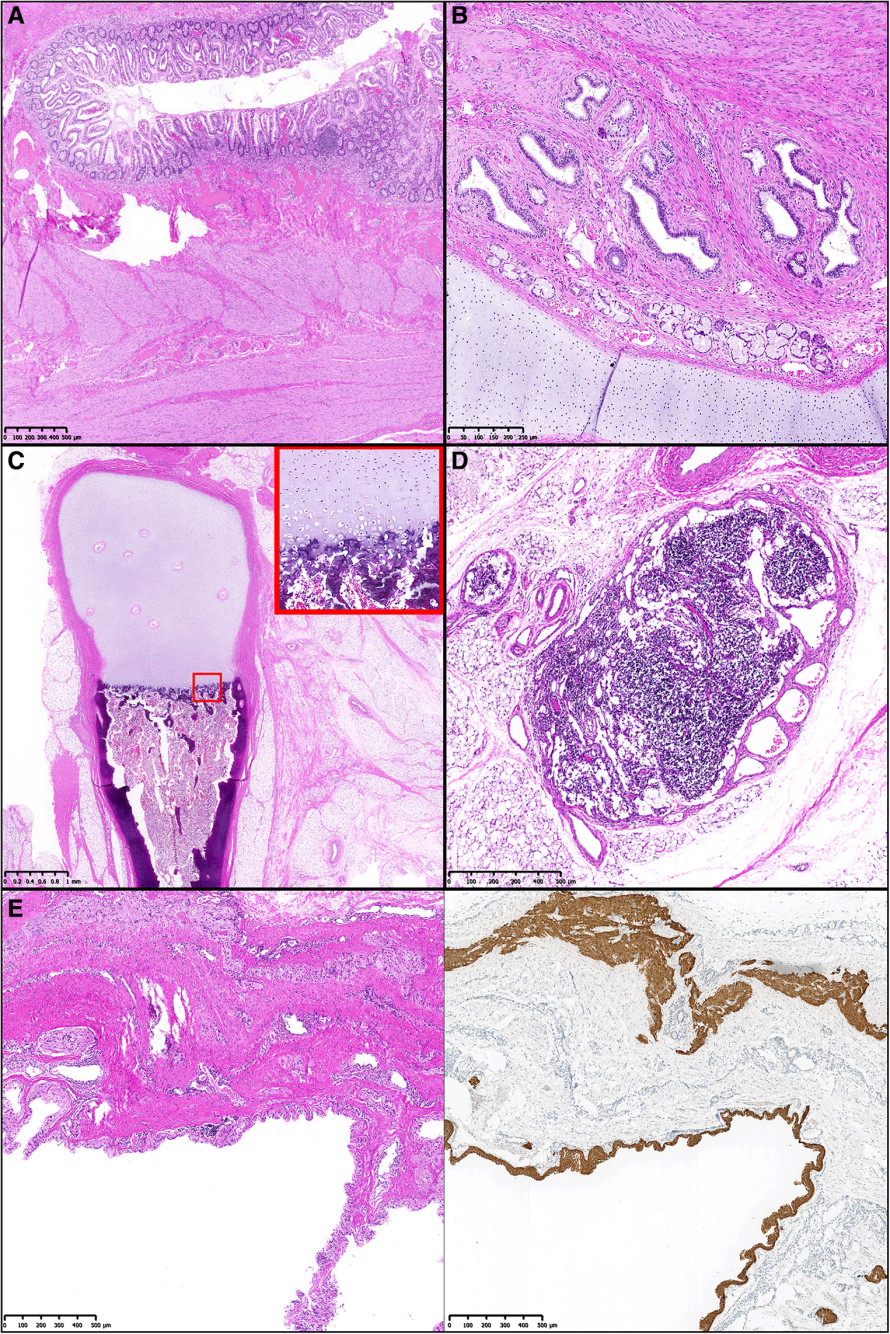
Microscopy with (**A**) regularly structured colonic wall with mucosa, submucosa, and bilayered muscularis propria. (HE). (**B**) Primitive but organized structure of a bronchus with bronchial mucosa, bronchial glands, and a hyaline cartilaginous brace (HE). (**C**) Section through primitive leg showing centrally located differentiated long tubular bone. This shows a regular anatomical architecture with organized enchondral ossification zone (inset) (HE). (**D**) Lymph node with regular architecture, capsule and feeding vessels (HE). (**E**) Section through primitive cerebral vesicle with thin lining by neuronal tissue. Additional neuronal tissue is found in the fibrosed sheath (HE staining, left). In the right half of the image, the central neuronal tissue is marked by GFAP immunohistochemistry staining.

In the histopathological preparation, the specimen presented as a pedunculated tumor that was completely covered by skin, showing multiple round nodular distensions (Figure 3). A rudimentary leg with a one-toed foot was found at one site, which histologically contained a long bone with inconspicuous endochondral and perichondral ossification. On section, there was a large cavity that histologically corresponded to a cerebral vesicle (Figure 4E). In addition, there were central cartilage fragments, possibly corresponding to primitive vertebrae, as well as only histologically recognizable tissue differentiations in the form of thyroid tissue, colon wall portions, and rudimentary bronchial structures (Figure 4B). Further circumscribed organ formations could not be detected.

The postoperative course including airway situation and the gradual normalization of oral feeding was always uneventful and the patient discharged on postoperative day 15.

Follow-up clinical controls and ultrasound investigations were performed after 1, 10 and 22 months. The ultrasound investigations showed stable, non-perfused residual cystic alterations in the submandibular area on the right side with a non-definable right submandibular gland on that side. Clinical examination of the thriving child was unremarkable except for an asymmetric soft palate and elongated uvula. Breathing, swallowing and voice production were not affected in any way.

## 3. Discussion

An epignathus is a rare condition caused by a palatal or pharyngeal mass that can continuously range in its entity from a mature or complex teratoma to a real fetus-in-fetu. Attachments of the masses to the oral cavity, the pharynx or the area of Rathke pouch with intracranial extension have been described (4, 7, 8). On the one end of the spectrum, teratoma are germ cell tumors generally comprised of differentiated tissues from all the three germ cell layers endoderm, ectoderm, and mesoderm (7, 9). They occur approximately in 1 in 4,000 live births and are more common in females (10). Compared to the sacrococcygeal or ovarial region, teratomas are less common in the head and neck region (11) and an occurrence in the oropharynx is even more uncommon (4). On the other extreme end of the continuous spectrum, fetuses-in-fetu are very rare endoparasites and mostly located in the retroperitoneum. A presentation as epignathus as in our case is exceedingly rare and has only been described in few individual reports (2, 5). Due to the rarity of fetuses-in-fetu with an incidence of less than 0.1 in 100,000 births, their broad range of possible manifestations as well as the blurred overlap with teratoma, there has been a great deal of dispute about their precise definition for many years (1, 4): In the past, a narrow definition of fetus-in-fetu included a mass with a longitudinal vertebral axis and differentiated organs around it (12, 13). Recently a broader definition of fetus-in-fetu has been proposed with at least one of the following criteria to be met: enclosure of the mass in a distinct sac, coverage with normal skin, grossly recognizable anatomical parts and attachment to the autosite via a peduncle containing larger blood vessels (4): In our case the latter 3 criteria are clearly fulfilled: Beside skin and a peduncle, a variety of well differentiated anatomic parts such as a rudimentary leg, a cerebral vesicle, thyroid tissue, colon wall portions and rudimentary bronchial structures could be identified (Figures 2–4). Additionally, the central cartilage fragments in our case could correspond to primitive vertebrae possibly indicating a longitudinal vertebral axis and hence even meeting the previously very narrow definition of a fetus-in-fetu.

Early prenatal diagnosis of an epignathus is vital for the outcome and prognosis. Depending on the size and the location of the attachment of the epignathus to its autosite, intrauterine complications ranging from preeclampsia to polyhydramnion have been reported (8, 14). Postnatally, a fatal or life-threatening airway obstruction or symptoms such as stridor, recurrent apnoea, and feeding difficulties have often been described (15). Hence pre- and immediate postnatal airway assessment by an experienced interdisciplinary team is crucial for the survival of the newborn. If necessary, an EXIT-Procedure (=Ex Utero Intrapartum Treatment first described by Norris in 1989 could be considered (16).

During EXIT procedure, intubation or tracheotomy might be performed while uteroplacental circulation with neonatal anaesthesia and controlled uterine hypotonia are maintained to avoid asphyxia (17, 18). Indications for EXIT might be cervical and pharyngeal masses, cervical vascular malformations, severe congenital diaphragmatic hernia or craniofacial abnormalities (19).

As for our case, diagnosis could be made early in gestation week 30 by intrauterine MRI scan, which confirmed a patent airway (Figures 1A,B). In particular, the nasopharynx was fluid filled and thus considered open (Figures 1A,B). Based on the radiologic MRI images and ultrasound findings, we decided against an EXIT procedure in our multidisciplinary team. Instead, we were planning a caesarian section, but under readiness for nasotracheal intubation of the autosite. As expected from the MRI images, the newborn presented with a stable airway and spontaneous breathing. With the absence of intracranial extension of the epignathus, a surgical excision via an enoral approach was performed 48 h after birth resulting in fast recovery and an excellent outcome in the 3 months follow-up examination. An increased incidence of anomalies such as a cleft palate and cardiac abnormalities have been associated with epignathus and should be considered in a thorough work up (6, 8). In our case however, clinical examination as well as a postnatal ultrasound and MRI scan of the neonate did not yield any significant anomalies except a small atrial septal defect further contributing to the excellent prognosis.

## 4. Conclusion

Epignathus can be caused by a continuous spectrum of anomalies ranging from mature teratoma to the exceedingly rare fetuses in fetu and many ENT surgeons might not encounter it at all in their careers. Regardless of the entity, by originating from the palate or pharynx, epignathus frequently causes life threatening airway obstruction. Hence early diagnosis and knowledge of the preoperative workup are essential to enable multidisciplinary management. Once the airway is stable, radical excision is the treatment of choice often resulting in a good clinical outcome and prognosis.

## Data Availability

The original contributions presented in the study are included in the article, further inquiries can be directed to the corresponding author.
